# Supplementary material for: Mutation Analysis of Pancreatic Juice and Plasma for the Detection of Pancreatic Cancer
Source: Int J Mol Sci. 2023 Aug 23;24(17):13116. doi: 10.3390/ijms241713116 (PMC10487634; doi:10.3390/ijms241713116)

Supplemental Table S1: in- and exclusion criteria per prospective cohort study.

Prospective cohort	Inclusion criteria	Exclusion criteria
**KRASPanc**	Patients who undergo an EUS for (suspected) PDAC either as part of a diagnostic process or fiducial placement prior to radiotherapeutic treatment.	Age <18 years
**PACYFIC**	Individuals with a suspected neoplastic pancreatic cyst (either newly or previously diagnosed, or previously operated upon) for which cyst surveillance is warranted, according to the treating physician.	Age <18 years, history of chronic pancreatitis, suspected pseudocyst (simple, thin-walled cyst that developed in the course of acute pancreatitis, as documented by sequential imaging studies), suspected serous cystadenoma (typical microcystic lesion with lobulated outlines, a calcified central scar and/or cyst fluid CEA levels < 5ng/ml), Von Hippel-Lindau disease, and limited life expectancy (<2 years).

Supplemental Table S2:

Panel	Samples tested	Genes (with hotspot coverage)
Oncomine Colon cfDNA Assay	Plasma and PJ samples of patients #1-4	*AKT1, BRAF, CTNNB1, EGFR, ERBB2, FBXW7, GNAS, KRAS, MAP2K1, NRAS, PIK3CA, SMAD4, TP53, APC*
in-house created pan-cancer AmpliSeq panel	Tissue of patients #1-6	*CDKN2A* (100%), *KEAP1* (100%), *PTEN* (100%), *STK11* (100%), *TP53* (100%).<br>Mutatie hotspots: *AKT1* (exon 3), *AKT2* (3), *AKT3* (2), *ALK* (20, 22-25), *APC* (16), *ARAF* (7), *BRAF* (11, 12, 14, 15), *CDK4* (2, 4, 7, 8), *CHEK2* (3, 4, 11, 12), *CTNNB1* (3, 7, 8), *DDR2* (14-19), *EGFR* (12, 18-21), *EIF1AX* (1, 2), *HER2* (8, 17-21), *ERBB3* (3, 6-10, 21, 23), *ESR1* (4, 5, 7, 8), *EZH2* (16), *FBWX7* (9, 10), *FGFR1* (4, 7, 12-14), *FGFR2* (7, 9, 12), *FGFR3* (7, 9, 14, 15), *FOXL2* (1), *GNA11* (4, 5), *GNAQ* (4, 5), *GNAS* (8, 9), *HRAS* (2-4), *IDH1* (4), *IDH2* (4), *JAK2* (14), *JAK3* (4, 16), *KIT* (8, 9, 11, 13-18), *KNSTRN* (1), *KRAS* (2-4), *MAP2K1* (1-6), *MET* (2, 14, 19, 20), *MTOR* (30, 39, 40, 43, 47, 53, 56, 57), *MYD88* (5), *NFE2L2* (2), *NOTCH1* (26, 27), *NRAS* (2-4), *OXA1L* (1), *PDGFRA* (12, 14, 18), *PIK3CA* (2, 5, 8, 10, 14, 21), *POLD1* (6, 8, 12, 15-17, 24), *POLE* (9-14, 21, 25), *RAC1* (2), *RAF1* (7), *RET* (11, 16), *RHOA* (2), *RIT1* (4, 5), *RNF43* (2-10), *ROS1* (36-41), *SF3B1* (14, 15), *SMAD4* (3, 9, 12).
Accel-Amplicon 57G Plus Pan-Cancer Profiling Panel	Plasma and PJ samples of patients #5-26	*ABL1, AKT1, ALK, APC, ATM, BRAF, CDH1, CDKN2A, CSF1R, CTNNB1, DDR2, DNMT3A, EGFR, ERBB2, ERBB4, EZH2, FBXW7, FGFR1, FGFR2, FGFR3, FLT3, FOXL2, GNA11, GNAQ, GNAS, HNF1A, HRAS, IDH1, IDH2, JAK2, JAK3, KDR, KIT, KRAS, MAP2K1, MET, MLH1, MPL, MSH6, NOTCH1, NPM1, NRAS, PDGFRA, PIK3CA, PTEN, PTPN11, RB1, RET, SMAD4, SMARCB1, SMO, SRC, STK11, TP53* (full exon coverage), *TSC1, TSC2, VHL*

Supplemental Table S3: The input volume (and yield of DNA) and responding Alu247/11p ratio, number of reads and coverage per biomaterial and patient.

Patient	Volume used PJ (uL)	Volume used plasma (uL)	DNA yield after isolation from PJ (ng)	DNA yield after isolation from plasma (ng)	Alu247/115 ratio in PJ	Alu247/115 ratio in Plasma	Used sequencing panel (both biomaterials)	PJ number of sequencing reads	Plasma number of sequencing reads	PJ coverage (%)	Plasma coverage (%)
HGD #1	500	1920	1368.00	87.30	NA	NA	Oncomine[1]	4811858	4987695	72.92	100.00%
PC #2	500	1930	1722.00	6.80	NA	NA	Oncomine[1]	6586294	4468304	95.83	95.83%
PC #3	500	1440	5390.00	9.90	NA	NA	Oncomine[1]	5008399	4007925	87.50	93.75%
PC #4	1000	1000	1404.00	4.70	NA	NA	Oncomine[1]	6987785	3957081	95.83	97.92%
Locall	720	2950	56.84	14.74	0.27	0.31	Swift[2]	2812510	4297124	90.96	95.48%
PC#6	700	2480	1298.50	6.86	0.61	0.26	Swift[2]	2578202	2823872	99.54	92.10%
PC #7	720	3280	7619.60	29.27	0.69	0.28	Swift[2]	2714452	2730770	99.51	96.35%
PC #8	720	2550	5192.00	19.24	0.71	0.17	Swift[2]	2612996	3367202	99.62	96.77%
PC #9	700	3200	3580.80	18.28	0.83	0.24	Swift[2]	2596180	2758396	99.56	96.92%
PC #10	720	2400	2119.70	13.10	0.52	0.22	Swift[2]	2648742	3380350	99.55	95.43%
PC #11	700	2850	1695.40	6.28	0.56	0.29	Swift[2]	2754432	2956516	99.55	88.47%
PC #12	700	3280	4264.00	22.76	0.83	0.28	Swift[2]	2694038	2871518	99.41	97.44%
PC #13	700	2100	854.40	25.92	0.04	0.25	Swift[2]	3450456	2950646	99.35	97.82%
PC #14	720	2570	2544.00	7.41	0.12	0.34	Swift[2]	2635210	2815604	99.72	90.89%
PC #15	700	2050	278.88	33.14	0.90	0.17	Swift[2]	2659392	2907950	99.59	97.82%
PC #16	1700	3100	8951.60	21.50	0.46	0.26	Swift[2]	1930452	2020394	99.57	89.09%
PC #17	380	3700	202.08	12.30	0.08	0.30	Swift[2]	1761106	1958542	99.05	83.86%
PC #18	600	3500	1310.40	36.60	0.84	0.11	Swift[2]	1939680	1968964	99.64	97.01%
PC #19	700	4080	19.63	20.30	0.18	0.24	Swift[2]	1672644	1672464	97.27	91.15%
PC #20	620	2950	2544.00	13.30	0.22	0.21	Swift[2]	1927712	1670568	99.74	89.52%
PC #21	700	4400	5472.00	21.60	0.24	0.32	Swift[2]	1651010	1928082	99.59	89.19%
PC #22	700	3450	1564.80	28.70	0.38	0.26	Swift[2]	1661178	2083404	99.52	91.41%
PC #23	620	3800	1107.40	16.30	0.19	0.28	Swift[2]	1860392	2071222	99.65	90.13%
PC #24	700	2580	3590.40	10.30	0.17	0.26	Swift[2]	1900044	2051182	99.70	87.19%
PC #25	730	2800	203.35	12.70	0.30	0.28	Swift[2]	1605274	1716840	99.64	91.10%
PC #26	700	3050	247.69	10.10	0.84	0.22	Swift[2]	1971610	1709320	99.47	86.84%

1. Oncomine Colon cfDNA Assay (Thermo Fisher Scientific, Waltham, MA); 2. Accel-Amplicon 57G Plus Pan-Cancer Profiling Panel (Swift Biosciences, Ann Arbor, MI, USA).

Supplemental Figure S1: H&E staining of tissue and biopsy material showing the cancer cellularity at time of resection for patient #1 with HGD (A-B) and patients #2 (C), #4 (D), #6 (E) with PC, and at time of biopsy for patient #5 (F) with PC. No slide was available of patient #3 at time of writing. HGD = high-grade dysplasia, PC = pancreatic cancer. The used magnification is 10x.

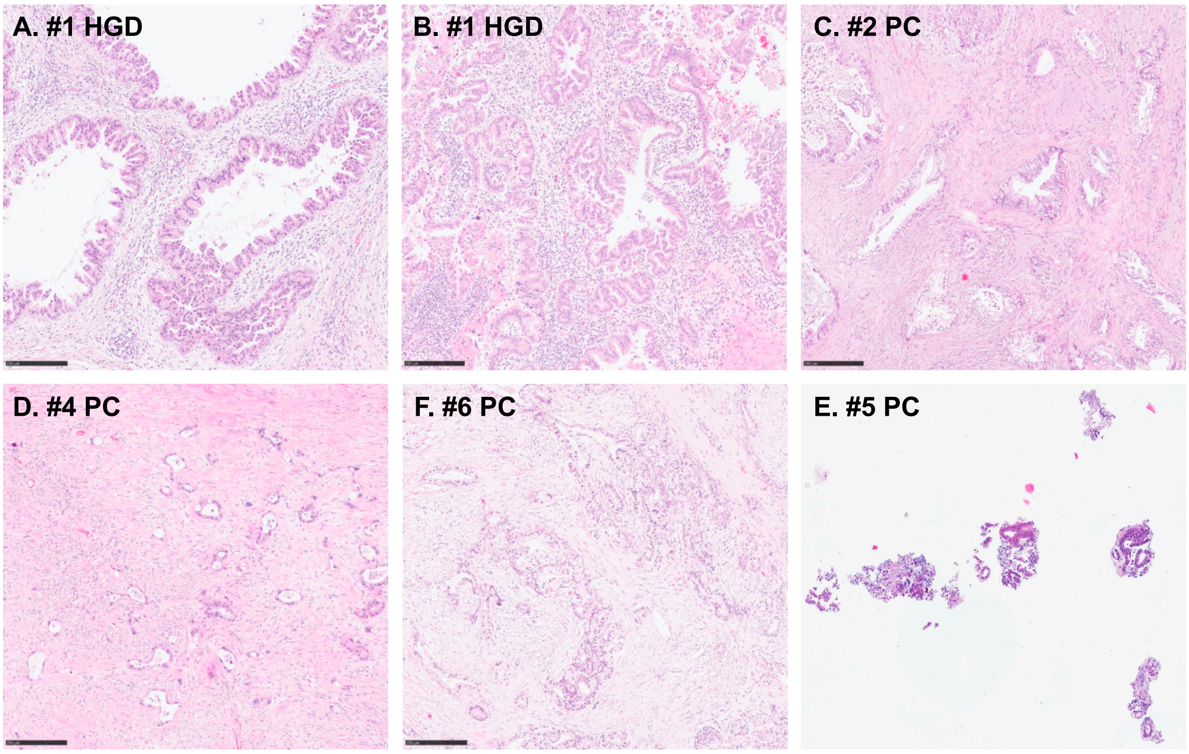

Supplement: Supplementary file 1 [file ijms-24-13116-s001.zip › ijms-2476005-supplementary.pdf]
